# Depletion of eIF4G from yeast cells narrows the range of translational efficiencies genome-wide

**DOI:** 10.1186/1471-2164-12-68

**Published:** 2011-01-26

**Authors:** Eun-Hee Park, Fan Zhang, Jonas Warringer, Per Sunnerhagen, Alan G Hinnebusch

**Affiliations:** 1 Laboratory of Gene Regulation and Development, Eunice K. Shriver National Institute of Child Health and Human Development, National Institutes of Health, Bethesda, Maryland 20892, USA; 2 Department of Cell and Molecular Biology, Lundberg Laboratory, University of Gothenburg, P.O. Box 462, S-405 30 Göteborg, Sweden

## Abstract

**Background:**

Eukaryotic translation initiation factor 4G (eIF4G) is thought to influence the translational efficiencies of cellular mRNAs by its roles in forming an eIF4F-mRNA-PABP mRNP that is competent for attachment of the 43S preinitiation complex, and in scanning through structured 5' UTR sequences. We have tested this hypothesis by determining the effects of genetically depleting eIF4G from yeast cells on global translational efficiencies (TEs), using gene expression microarrays to measure the abundance of mRNA in polysomes relative to total mRNA for ~5900 genes.

**Results:**

Although depletion of eIF4G is lethal and reduces protein synthesis by ~75%, it had small effects (less than a factor of 1.5) on the relative TE of most genes. Within these limits, however, depleting eIF4G narrowed the range of translational efficiencies genome-wide, with mRNAs of better than average TE being translated relatively worse, and mRNAs with lower than average TE being translated relatively better. Surprisingly, the fraction of mRNAs most dependent on eIF4G display an average 5' UTR length at or below the mean for all yeast genes.

**Conclusions:**

This finding suggests that eIF4G is more critical for ribosome attachment to mRNAs than for scanning long, structured 5' UTRs. Our results also indicate that eIF4G, and the closed-loop mRNP it assembles with the m^7 ^G cap- and poly(A)-binding factors (eIF4E and PABP), is not essential for translation of most (if not all) mRNAs but enhances the differentiation of translational efficiencies genome-wide.

## Background

Translation of most mRNAs in eukaryotic cells occurs by a scanning mechanism wherein the small (40S) ribosomal subunit recruits methionyl initiator tRNA (Met-tRNA_i_^Met^) in a ternary complex with eIF2-GTP, in a reaction stimulated by other eIFs, and the resulting 43S pre-initiation complex (PIC) binds near the m^7 ^G-cap structure of the mRNA to assemble the 48S PIC. Attachment of the 43S complex at the mRNA 5' end is stimulated by the eIF4F complex, comprised of cap-binding protein eIF4E, the scaffold subunit eIF4G, and the DExD/H-box helicase eIF4A, which is thought to provide a single-stranded region in the mRNA for recruiting the ribosome. Binding sites in eIF4G for either eIF3 (in mammals) or eIF5 and eIF1 (in yeast) are thought to facilitate recruitment of the 43S PIC to eIF4F bound at the cap structure. eIF4G also harbors a binding site for the poly(A)-binding protein (PABP) that, together with an RNA binding domain in the middle region of mammalian eIF4G, increases the stability of eIF4F binding to the mRNA 5' end and also mediates circularization of mRNA in the activated eIF4F·mRNA·PABP mRNP [1-3].

In addition to stimulating recruitment of the 43S PIC to the mRNA 5' end, there is evidence that the ATP-dependent RNA helicase activity of eIF4A facilitates ribosomal scanning through secondary structures in the 5' UTR to enhance recognition of the AUG start codon [4,5]. However, other DExD/H helicases have been implicated in scanning through long or structured 5' UTRs, including Ded1/DDX3 in yeast [6] and DHX29 in mammals [7], and it is uncertain whether eIF4A and its binding partners in eIF4F are critically required for scanning. In fact, 43S recruitment and location of the start codon has been reconstituted in vitro for an artificial mRNA with an unstructured 5' UTR in the absence of eIF4F, eIF4A, eIF4B, and ATP, requiring only the eIF2·GTP·Met-tRNA_i _^Met ^ternary complex, eIF3, eIF1, and eIF1A [5]. Hence, it is possible that native mRNAs devoid of stable structures in the 5'UTR could be translated at relatively high efficiencies in the absence of eIF4F. Indeed, we showed previously that genetically depleting eIF4G from yeast cells reduces general translation initiation but does not impair 48S PIC formation in vivo by two native mRNAs (*RPL41A *and *MFA2*) [8].

Based on its presumed functions in mRNA activation and scanning, it is generally assumed that eIF4F plays an important role in determining the relative efficiencies of translation among the repertoire of cellular mRNAs and, hence, is a key factor in translational control of gene expression [9]. We examined this hypothesis in yeast by measuring the effect of genetically depleting eIF4G from yeast cells on translational efficiencies of mRNAs genome-wide. The depletion of eIF4G was very effective and it reduced protein synthesis rates by a factor of ~3, leading to cell growth arrest. Surprisingly, however, the translational efficiencies of most mRNAs were not substantially affected by eIF4G depletion. An intriguing consequence of a strong reduction in eIF4G levels was to narrow the range of translational efficiencies genome-wide by reducing the translation of many mRNAs with higher than average translational efficiencies in wild-type cells while increasing the translation of different mRNAs that are normally translated with lower than average efficiencies. Our findings suggest that eIF4G is not essential for translation of any mRNAs in yeast cells, but it enhances the differentiation of translational efficiencies among cellular mRNAs.

## Results

### Depletion of eIF4G1 in cells lacking eIF4G2 evokes a marked decrease in the rate of translation initiation in vivo

To examine the consequences for global translation of eliminating both isoforms of eIF4G, we employed a strain deleted of the chromosomal gene encoding eIF4G2 (*tif4632Δ*) and harboring a temperature-sensitive degron allele [10] of the gene encoding eIF4G1 (*tif4631-td*). The *tif4631-td *allele encodes ubiquitin and a thermolabile dihydrofolate reductase moiety fused to the N terminus of eIF4G1, expressed from a copper-dependent promoter, and is integrated into the chromosome in a manner that disrupts the resident wild-type (WT) *TIF4631 *allele [8]. The strain also contains a galactose-inducible form of the gene encoding the ubiquitin ligase (Ubr1) required for proteasomal degradation of degron-tagged proteins by the "N-end rule" pathway [11]. Shifting cells from medium containing copper and raffinose (as carbon source) at 25°C to medium containing galactose and raffinose but lacking copper at 36°C represses new synthesis and triggers proteasomal degradation of the existing degron-tagged eIF4G1-td protein. We showed previously that under non-permissive conditions this degron mutant cannot form colonies from single cells, exhibits a strong reduction in doubling time within 2 h, and essentially ceases growth and division by 8 h after the shift to non-permissive conditions. This growth arrest can be reversed by shifting cells back to permissive conditions [8].

**Figure 1:**
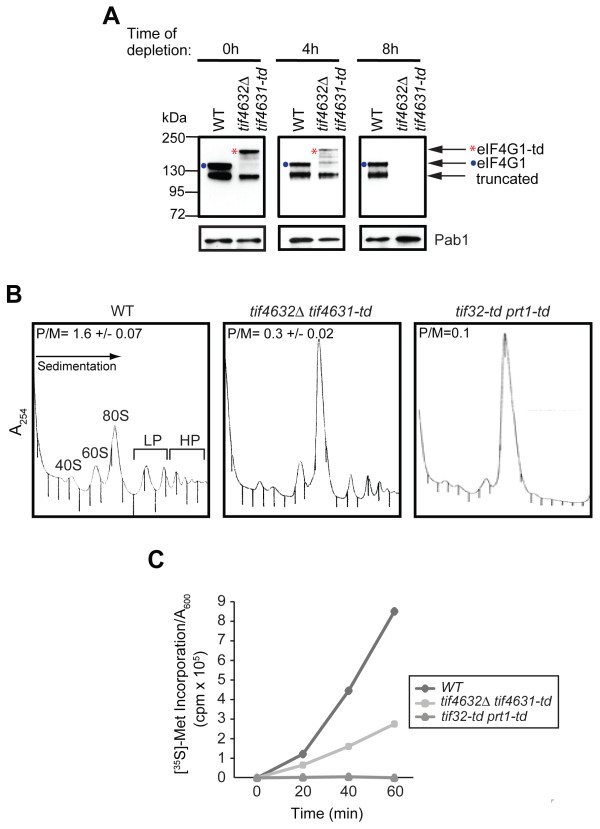
**Depletion of degron-tagged eIF4G1 in a mutant lacking eIF4G2 reduces, but does not abolish, translation initiation**. **(A) **Degron-tagged eIF4G1 is undetectable after 8 h in nonpermissive conditions. Wildtype (WT) strain YAJ3 and *tif4631-td tif4632Δ*degron mutant YAJ41 were grown under permissive conditions to A_600 _of 0.3 and shifted to nonpermissive conditions for 4 h or 8 h. WCEs were prepared and subjected to Western blot analysis to monitor expression levels of eIF4G1 and Pab1 (examined as a loading control). **(B) **Polysome content is reduced in eIF4G-depleted cells. WT strain YAJ3 and the indicated degron mutants (YAJ41 and YAJ34) were grown under permissive conditions and shifted to nonpermissive conditions for 8 h. Following treatment with cycloheximide, WCEs were prepared and resolved by sedimentation through sucrose gradients. Gradient fractions were scanned at A_254 _to determine polysome/monosome ratios (P/M, mean+S.E.M., n = 4). The P/M ratio in the *tif32-td prt1-td *strain is an average from two independent gradients. The positions of the 40S and 60S ribosomal subunits, 80S monosome, light polysomes (LP), and heavy polysomes (HP) are indicated. **(C) **The rate of protein synthesis is reduced in eIF4G-depleted cells. The strains from (B) were cultured as described there and labeled with [^35 ^S]-methionine. Acid-insoluble radioactivity, normalized for the A_600 _of the cells, was measured in aliquots of the cultures collected at the indicated times.

Consistent with our previous results, incubation for 8 h under non-permissive conditions was required to deplete eIF4G1-td in whole cell extracts (WCEs) below the detection limit of Western analysis (Figure 1A). Note that both the wild-type (WT) and mutant WCEs appear to contain an N-terminally truncated form of eIF4G1 that migrates more rapidly than either the WT or degron-tagged full-length proteins (Figure 1A). (If truncated at the C-terminus, the degron-tagged protein would be larger than the cognate truncation of WT eIF4G1.) Because this truncation is subject to degradation in the degron mutant, but necessarily lacks the N-terminal modifications necessary for N-end rule degradation, it is likely generated from the full-length proteins in vitro following cell lysis.

After 8 h of depletion, the degron mutant exhibits the expected reduction in total polysomes and commensurate increase in 80S monosomes, leading to a decreased ratio of polysomes to monosomes (P/M) by a factor > 5 compared to the P/M ratio for the WT strain under the same conditions (Figure 1B). This is the stereotypical consequence of selective impairment of translation initiation, involving a decrease in new initiation events, "run-off" of elongating ribosomes from existing polysomes, and subsequent accumulation of excess free subunits as 80S couples. Note that depletion of two essential subunits of the eIF3 complex, in a separate mutant expressing degron-tagged forms of these proteins [8], evokes a more complete polysome run-off than observed in the eIF4G1-td mutant (Figure 1B). Consistent with the polysome profiles, the rate of total protein synthesis, measured by incorporation of radioactive methionine into acid-insoluble material, was reduced in the eIF4G1-td mutant to ~30% of the WT value (Figure 1C) after 8 h in the non-permissive condition, whereas the eIF3 degron mutant displayed no detectable [^35 ^S]-Met incorporation under these conditions (Figure 1C). Thus, in accordance with our previous conclusions, depletion of eIF4G1 in cells lacking eIF4G2 leads to a marked reduction in the rate of translation initiation, but one less severe than that provoked by a comparable depletion of eIF3 subunits [8]. (Henceforth, rather than referring to depletion of eIF4G1 in the degron mutant lacking eIF4G2, we describe it more simply as depletion of eIF4G.)

### Depletion of eIF4G narrows the range of mRNA translational efficiencies genome-wide

Although a significant level of translation continues following the extensive depletion of eIF4G in the degron mutant (Figure 1), it was possible that translation of some mRNAs would be greatly diminished while translation of others would continue relatively unaffected or even increase. To address this possibility, we determined the effect of depleting eIF4G on the translational efficiencies of mRNAs genome-wide. To this end, we conducted microarray analysis on RNA isolated from the "heaviest" polysomes, containing 4 or more elongating 80S ribosomes per mRNA (Figure 1B, "HP" fractions), and also total RNA from WCEs, from both degron mutant and WT cells cultured for 8 h under non-permissive conditions. Translational efficiencies (TEs) were calculated for each gene as the ratio of hybridization intensities on microarrays probed with cDNAs produced from HP versus total RNA samples. It should be noted that equal amounts of cDNA are used to probe each microarray and the intensities are scaled so that each array has approximately the same average value. This normalization will diminish the effect of reduced polysome abundance in the eIF4G mutant versus WT cells. The total amount of mRNA could also decline in the mutant owing to reduced transcription or increased mRNA turnover accompanying diminished translation, which would offset the effect of decreased polysome abundance on the calculated translational efficiencies. Hence, comparing TE values can indicate absolute differences in translational efficiency between two genes in the same strain, but it reveals only relative differences in efficiency for a given gene between two strains.

**Figure 2:**
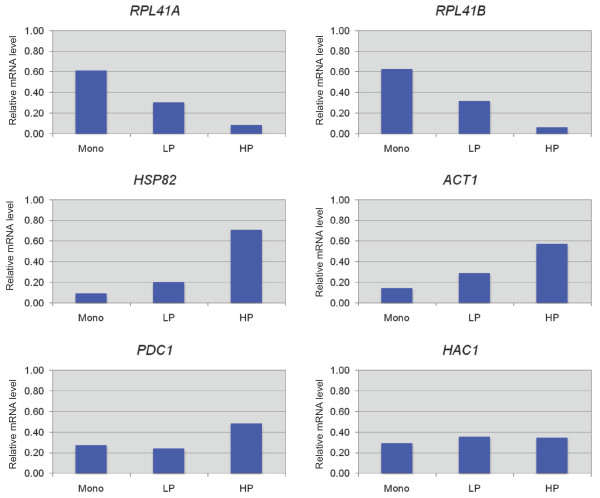
**Analysis of polysome distributions of selected mRNAs**. WT strain YAJ3 was grown under non-permissive conditions as described above and WCEs were resolved by sedimentation through sucrose gradients as in Figure 1B. The mRNAs indicated above each panel were quantified by qRT-PCR in 1 μg of RNA from the 80S (Mono), LP, and HP fractions. The resulting values were normalized to the levels of 18S rRNA quantified in the same samples and then multiplied by a factor corresponding to the proportion of total A_280 _units in the gradient present in the cognate fractions (80S, LP, or HP). The results for each fraction are plotted as a proportion of the total amount present in all three fractions combined.

As a quality control for the polysomal fractionation and mRNA extraction procedures, we first analyzed the distribution of several mRNAs among heavy polysomes, light polysomes (LP, 2-mers and 3-mers), and 80S monosomes using real-time RT-PCR to quantify mRNA concentrations (see Materials and Methods). The distributions of *RPL41A *and *RPL41B *mRNAs were examined because their coding sequences, of only 78 nt [12], are large enough to accommodate only two translating 80S ribosomes [13], and at the average ribosome density for yeast mRNAs they should generally contain only one translating 80S ribosome at a time [14]; hence, the majority of these two mRNAs should occur in the 80S monosome fraction. The distributions of *RPL41A *and *RPL41B *mRNAs observed for WT cells were highly similar to one another and displayed the expected preponderance of mRNA in the 80S fractions and smaller proportions in the LP fractions (Figure 2, top 2 panels). (The minor signals for these mRNAs in the HP fractions could arise from a small degree of contamination from the Mono or LP fractions during collection of the gradient fractions, or from aggregation of polysomes.) By contrast, the *HSP82, PDC1*, and *ACT1 *mRNAs were most abundant in the HP fractions and least abundant in the 80S or LP fractions, whereas *HAC1 *mRNA showed relatively equal abundance in all three fractions (Figure 2). These findings are in accordance with previous polysomal profiling of these four mRNAs [14].

For microarray analysis, three biological replicates were examined (designated projects I, II, and III), representing HP and total RNA preparations from three independent pairs of WT and mutant cultures. Cy3-labeled cDNAs were generated from the 3 HP and 3 total RNA samples prepared for each strain and the resulting 12 sets of cDNAs were used to probe three (technical) replicate whole-genome microarrays, containing multiple 60-mer oligonucleotides for each gene (36 arrays in total). The "normalized gene expression summary values" were calculated for each gene from the data obtained from the three technical replicates and used to calculate the translational efficiency (TE) of each gene as the ratio of the intensity values for HP to total RNA (HP/T) for each project (see Additional file 1).

**Figure 3:**
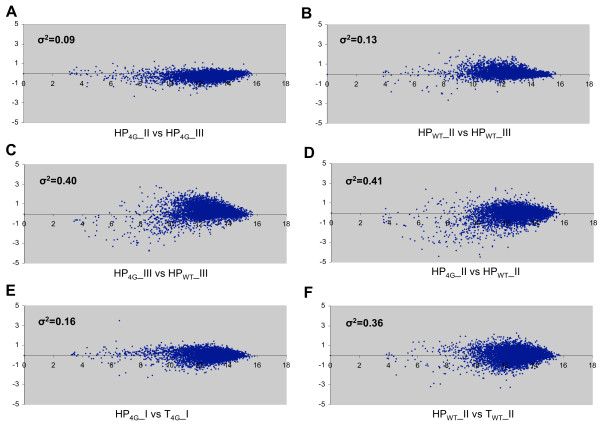
**MA plots of microarray data**. **(A-F) **Plots were constructed from microarray data for heavy polysomal (HP) or total (T) RNA samples obtained for WT or degron mutant (4G) cells in projects I, II, or III. *M *is calculated for each gene as log_2 _(I_1_)-log_2 _(I_2_), where I_1 _and I_2 _are the signal intensities from arrays 1 and 2, and quantifies the ratio of intensities between the two microarrays. *A *is calculated as 0.5 (log_2 _(I_1_)-log_2 _(I_2_)), and quantifies the average intensity for each gene in the two arrays. Plotting *M *against *A *reveals the differences in intensities between the two arrays as a proportion of the average intensity for each gene. The variance (***σ^2^***) of *M *is shown in each plot.

We first constructed MA plots [15] to evaluate the reproducibility of mRNA intensities measured for the biological replicates of each strain. Such plots display the ratios of mRNA intensities between two arrays (quantified by *M*) as a function of the average intensities of the mRNAs (quantified by *A*). The variance (***σ^2^***) of *M *provides a measure of the range of intensity differences between two arrays across the genome. Representative MA plots are shown in Figures 3A-B, and the variances are summarized in Table S1 (see Additional file 2). The comparisons of biological replicates from the same strain yielded relatively low ***σ^2 ^***values for both HP and total RNA samples, that compare favorably with ***σ^2 ^***values reported previously for biological replicates of polysomal RNA [15]. We also used MA plots to compare the intensities of HP or total mRNAs between mutant and WT cells, and the variances in these plots were substantially higher than the corresponding values for replicates from the same strain (Table S1 in Additional file 2 and Figure 3C-D). These latter plots indicate significant differences in the intensities of both total and HP mRNAs between mutant and WT cells for a large fraction of the genome.

**Table 1:** Numbers of genes with higher or lower than mean translational efficiencies (TE) in wild-type and eIF4G mutant cells^1^

	(1) TE ≥ 1.5	(2) TE ≥ 2.0	(3) TE ≤ 0.67	(4) TE ≤ 0.5
(1)Wild-type	968	223	917	269
(2) eIF4G mutant	358	19	507	118

^1 ^Mean translational efficiencies were calculated by averaging TE values from all three projects for each gene

Finally, we constructed MA plots to quantify the differences in mRNA abundance in polysomes versus total mRNA, to visualize the variation in translational efficiency (HP/T) across the genome for each strain. Interestingly, the ***σ^2 ^***values for the HP:T intensity ratios are ~2-fold higher for WT than for mutant cells (Table S1 in Additional file 2), as illustrated in Figure 3E-F. This was the first indication that the breadth of translational efficiencies (HP/T values) across the genome is reduced by depletion of eIF4G.

**Figure 4:**
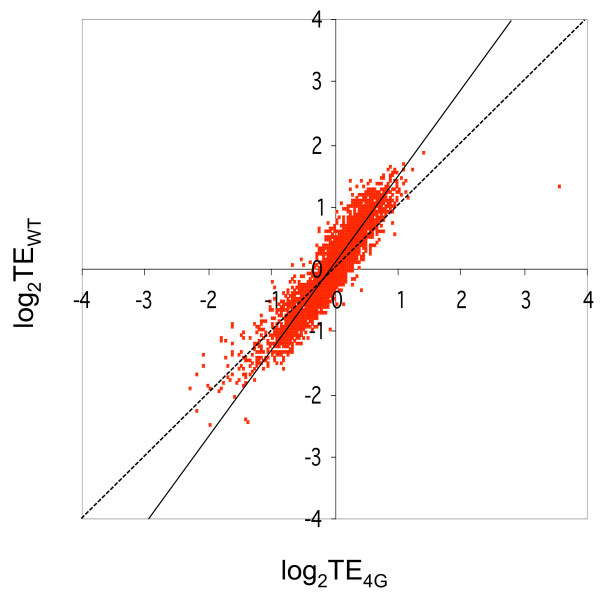
**Comparison of translational efficiencies genome-wide between WT and eIF4G degron mutant cells**. The log_2 _(TE) values determined from the mean TE values calculated using data from all three biological replicates for WT cells are plotted against the corresponding log_2_(TE) values for eIF4G mutant cells. The solid line is the experimentally determined regression line; the dotted line is the theoretical regression line in the hypothetical situation where TE values for all genes are identical between mutant and WT cells.

To depict graphically the population of mRNAs that are translated with relatively higher or lower efficiencies in WT versus mutant cells, we constructed scatter plots of HP/T ratios (TE values) for WT versus mutant mRNAs using the mean TE values calculated by averaging data from all three biological replicates (Figure 4). The regression line of the scatter plot has a slope significantly larger than unity (1.30), which indicates that mRNAs with greater than average TE in WT (TE_WT_) (points above the x-axis) tend to be translated at relatively lower efficiencies in the mutant cells. Moreover, mRNAs with lower than average TE in WT (points below x-axis) tend to be translated relatively better in the mutant. Considering the 2934 genes with TE values larger than the genome average in wild-type cells, the TE_WT_/TE_4G _ratio (averaged over all three projects) is 1.14. For the remaining genes with TE values smaller than the genome average, the mean TE_WT_/TE_4G _ratio is 0.91. As a consequence of these trends, there is a narrower range of translational efficiencies at both ends of the spectrum, in mutant versus WT cells.

This last conclusion was further supported by tabulating the numbers of mRNAs with TE values above or below unity between mutant and WT cells. In WT, 968 mRNAs have mean TEs ≥ 1.5, and 223 mRNAs have mean TE values ≥ 2.0 (Table 1, row 1, columns 1-2). In the mutant cells these gene categories are much smaller (Table 1, cf. rows 1-2, columns 1-2), indicating that a considerably smaller proportion of mRNAs have higher than average translational efficiencies in the mutant cells. A similar trend applies to mRNAs with relatively low TE values (Table 1, columns 3-4). Thus, the proportions of mRNAs translated with either higher or lower than average translational efficiencies are reduced on depletion of eIF4G.

The fact that the range of translational efficiencies is restricted by eIF4G depletion implies that eIF4G contributes to the higher than average TE values for the most efficiently translated mRNAs in WT cells. To verify this deduction, we determined the proportion of the mRNAs with TE_WT _values ≥ 1.5 that are translated more efficiently in WT versus mutant cells, ie. TE_WT _≥ 1.5 ∩ TE_WT _> TE_4G_. This condition holds for > 97% of the 968 mRNAs with TE_WT _≥ 1.5. A similar conclusion emerged for the 917 mRNAs with TE_WT _≤ 0.67, of which ~90% are translated less efficiently in WT than in mutant cells (TE_WT _≤ 0.67 ∩ TE_WT _> TE_4G_). This last comparison confirms that the least efficiently translated group of mRNAs in WT cells owe their relatively low TE values, at least partly, to the presence of eIF4G function. Below, we consider different mechanisms that could account for this negative effect of eIF4G on translational efficiency.

### Only a small proportion of genes exhibit substantially altered translational efficiencies on depletion of eIF4G

**Figure 5:**
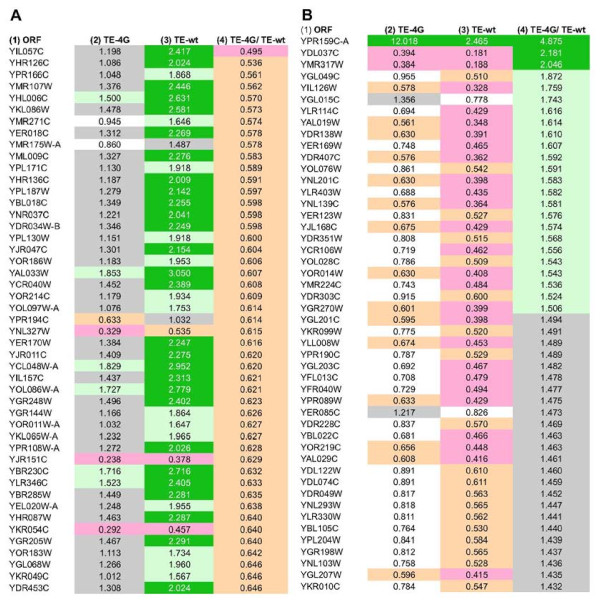
**Evidence that eIF4G contributes to both higher than average and lower than average translational efficiencies of specific genes**. **(A) **The mean TE values averaged from all three projects for the WT (TE-wt) and eIF4G degron mutant (TE-4G) are listed for 47 of the 94 mRNAs that exhibit mean TE_4G_/TE_WT _ratios of ≤ 0.71. TE values are highlighted in color as follows: ≥2, dark green; ≥ 1.5 and < 2, light green; ≥ 1.0 and < 1.5, grey; ≤ 0.5, pink; ≤ 0.67 and > 0.5, orange; > 0.67 and < 1.0, white. **(B) **The mean TE values averaged from all three projects for the WT (TE-wt) and eIF4G degron mutant (TE-4G) are listed for 48 of the 99 mRNAs that exhibit mean TE_4G_/TE_WT _ratios of ≥1.4, highlighted as in (A).

We focused next on the particular mRNAs whose translational efficiencies differ the most between mutant and WT cells (i.e. with TE_4G_/TE_WT _ratios that deviate the most from unity.) Because the difference in TE between mutant and WT cells is modest for the majority of mRNAs, coupled with the experimental variability in TE values calculated from the different projects, there is a small fraction of genes for which the difference between mean TE_4G _and TE_WT _values calculated from all three projects is statistically significant. We were able to identify 94 mRNAs (1.6% of the 5868 ORFs) that exhibit mean TE_4G_/TE_WT _ratios of ≤ 0.71 and for which the mean TE_4G _value differed from the mean TE_WT _value in all three projects with a P-value ≤ 0.1 in a two-tailed Student's t-test, of which 61 mRNAs differed with a P-value of ≤ 0.05 (Additional file 1). A subset of these 94 mRNAs are listed in Figure 5A (column 1), sorted on the mean TE_4G_/TE_WT _values (column 4). Note that most of these mRNAs exhibit relatively high TE values in WT cells (column 3) but display TEs in the mutant closer to unity (column 2). Thus, these genes all exhibit higher than average translational efficiencies in WT cells that are reduced in the mutant to values closer to the genome-average TE value (1.05 ± 0.004 in the mutant).

We similarly identified 99 mRNAs exhibiting a higher translational efficiency in the mutant versus WT, with mean TE_4G_/TE_WT _ratios ≥1.4 and for which the difference between the mean TE_4G _and TE_WT _values was significant at P ≤ 0.1, of which 46 differed with a P-value of ≤ 0.05 (Additional file 1). As illustrated in Figure 5B, the majority of such mRNAs exhibit lower than average translational efficiencies in WT cells with TE_WT _values ≤ 0.5 (column 3), but efficiencies in the mutant that are closer to the genome-average TE value (Figure 5B, column 2). Thus, their relatively low TE values in WT cells are increased on depletion of eIF4G in the mutant. These comparisons support the conclusion that eliminating eIF4G narrows the range of translational efficiencies at both ends of the spectrum.

In an effort to validate the microarray measurements of TE values, we conducted real-time qRT-PCR analysis of particular mRNAs in the polysomal and total RNA preparations used to produce the Cy3-cDNAs for probing microarrays. We analyzed a set of 28 genes, most belonging to the two groups of genes just described with mean TE_4G _values that are higher or lower than the cognate mean TE_WT _values by a factor of 1.4 or more. As shown in Figure S1 (Additional file 2), the mRNAs identified by microarray analysis with mean TE_4G_/TE_WT _ratios ≥1.4 displayed corresponding TE_4G_/TE_WT _ratios measured by qRT-PCR that were significantly greater than those for mRNAs with mean TE_4G_/TE_WT _values of ≤ 0.71 in the microarray analysis. Thus, it appears that the microarray analysis reliably identified two groups of genes that are affected oppositely by depletion of eIF4G.

### Characteristics of genes exhibiting altered translational efficiencies on depletion of eIF4G

**Figure 6:**
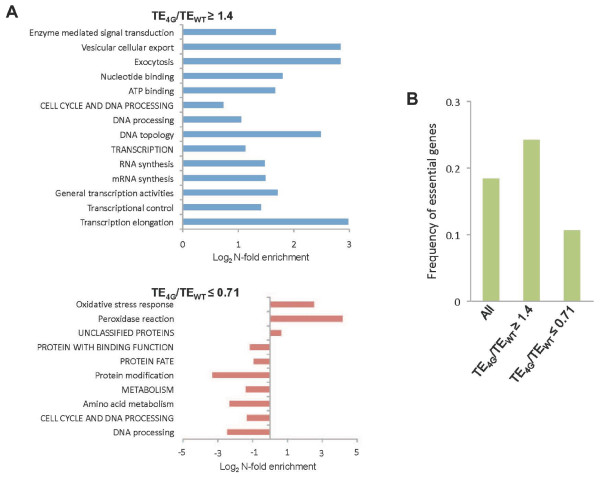
**Characteristics of genes exhibiting altered translational efficiencies on depletion of eIF4G.(A)** Cellular functions enriched among mRNAs translated relatively better (TE_4G_/TE_WT _≥1.4) or relatively worse (TE_4G_/TE_WT _≤ 0.71) on eIF4G depletion. The degree of enrichment (Log_2 _N-fold enrichment) as compared to the frequency among all tested genes for significantly enriched or underrepresented functional categories (P < 0.05, Fisher's exact test, Bonferroni correction) is shown. Functional enrichment was analyzed using the MIPS Funcat system (http://mips.helmholtz-muenchen.de/genre/proj/yeast/Search/Catalogs/catalog.jsp) **(B) **Frequency of essential genes within the complete set of tested genes (All) and among genes with TE_4G_/TE_WT _≥1.4 or TE_4G_/TE_WT _≤ 0.71. The frequency of essential genes in the latter two categories deviate significantly from the frequency for all tested genes (Fisher's exact test; P < 0.05).

We wished next to determine whether the genes that displayed the largest differences in translational efficiencies between mutant and WT cells tend to be involved in common biological processes. To this end, we conducted a gene ontology analysis using the MIPS Funcat system (http://mips.helmholtz-muenchen.de/genre/proj/yeast/Search/Catalogs/catalog.jsp), which determines whether genes of interest are significantly enriched in particular cellular functions. Analysis of the 99 genes with TE_4G_/TE_WT _≥1.4, which are translated relatively better on eIF4G depletion, revealed that they were enriched for genes with specific cellular functions (P < 0.05, Fisher's exact test, Bonferroni correction) (Figure 6A). This encompasses genes involved in multiple key aspects of transcription and RNA processing, such as the core transcriptional machinery (*RPB2*), histone assembly or modification (*SPT16*, *SET2*), transcription factors of the TOR growth control pathway (*RTG3*, *SFP1*), and components of the THO mRNA export complex (HPR1, THO2), as well as DNA processing components, especially as involved in control of DNA topology (*MCM6*, *STH1*, *TOF2*). Similarly enriched were genes involved in plasma membrane related trafficking, both endocytosis (*ALY2*, *MON2*) and exocytosis (*AVL9*, *CHS5*). Many of these processes correspond to key housekeeping functions, explaining the enrichment for essential genes (P < 0.05) evident in Figure 6B. Whether the increased translational efficiency of these housekeeping genes following depletion of eIF4G is a consequence of relief from translational repression exerted by eIF4G, or if it corresponds to a more general cellular effort to counter the effects of loss of eIF4G, is not clear. Notably, the 94 genes translated less efficiently following depletion of eIF4G tended not to encompass essential genes (Figure 6B), and several housekeeping processes, such as DNA processing and protein modification were underrepresented in this group (Figure 6A). In contrast, it was enriched for genes involved in oxidative stress response, especially components of the cellular peroxidase/thioredoxin systems, such as *GPX1*, *HYR1*, *TRX3*, *SRX1 *and *TSA2*. These findings suggest that under conditions of eIF4G down-regulation, a select group of mRNAs whose products function in housekeeping processes such as transcription and DNA processing, are translated relatively better than all other mRNAs; whereas a group of non-essential genes involved in cellular energy production are translated relatively worse.

**Figure 7:**
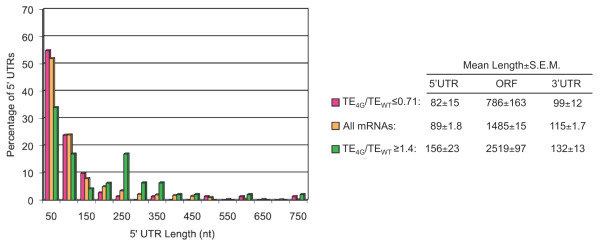
**Comparison of 5' UTR, ORF, and 3'UTR length distributions of genes with TE values in the eIF4G mutant substantially higher or lower than in WT cells**. The 5' UTR length distributions were compiled for the 70 genes that exhibit mean TE_4G_/TE_WT _ratios of ≤ 0.71 in projects I-III (red), for the 47 genes with mean TE_4G_/TE_WT _ratios ≥1.4 in projects I-III (green), and for all 4149 yeast genes (orange), for the subset of genes whose 5' UTR features were compiled by Lawless et al. (2009). ORF and 3'UTR lengths were calculated using Table S3 of Nagalakshmi et al [35]. Mean (+/- S.E.M.) values are listed for each group.

Given the reported involvement of eIF4G in activating mRNAs for recruitment of the 43S PIC and scanning the 5'UTR, we examined the two sets of genes with significantly altered TE_4G_/TE_WT _ratios to determine whether they exhibit atypical 5'UTR lengths or secondary structures. We employed the database of 5'UTR lengths for 4149 yeast ORFs from Lawless et al (2009) compiled from results of genome-wide studies of 5' transcription start sites. Interestingly, for the 47 genes with TE_4G_/TE_WT _≥1.4 whose features were compiled by Lawless et al, the mean 5' UTR length is 156 ± 23 nt, which is ~1.75 fold greater than the average 5'UTR length of 89 ± 1.8 nt for all 4149 genes in the database (P-value of 0.0001) [16]. For the 70 genes with TE_4G_/TE_WT _values ≤ 0.71, the mean 5' UTR length is 82 ± 15 nt, significantly smaller than that determined for the genes with TE_4G_/TE_WT _≥1.4 (P-value of 0.006) but not significantly different than the mean value for all mRNAs. The enrichment for long 5'UTR lengths for genes with TE_4G_/TE_WT _≥1.4 is evident in Figure 7, where their length distribution is compared to that of all 4149 5'UTRs (green versus yellow bars). Thus, the fraction of genes exhibiting a relative increase in TE in the mutant have a significantly longer than average 5'UTR, whereas those exhibiting a relative decrease in TE on eIF4G depletion have a nearly typical length distribution. Thus, the class of mRNAs most dependent on eIF4G exhibit the comparatively short 5'UTR lengths characteristic of the majority of yeast mRNAs.

Using the computer program RandFold to predict folding of 5'UTRs, Lawless et al reported that the vast majority of yeast 5' UTRs appear not to be strongly folded, with only 20 5'UTRs showing low minimum free energies (MFEs) of folding with an associated P-value of < 0.005. None of these mRNAs was found among the genes showing the greatest reductions in TE in the mutant versus WT (TE_4G_/TE_WT _≤ 0.71). In fact, four of these 20 mRNAs, all containing long 5'UTRs (> 235 nt) with strong predicted secondary structures, appear to be translated more efficiently on depletion of eIF4G, showing mean TE_4G_/TE_WT _ratios of 1.62 ± 0.46 (*YDL122W*), 1.37 ± 0.23 (*YGL215W*), 1.24 ± 0.05 (*YGL008C*), and 1.24 ± 0.03 (*YDL224C*). These results do not support the possibility that the translation of mRNAs with highly stable secondary structures in their 5'UTRs would be strongly enhanced by eIF4G.

It has been reported that mammalian eIF4G plays a critical role in the ability of post-termination 40S subunits to resume scanning following translation of a short uORF [17]. Hence, we asked whether the genes whose translation is relatively lower in the mutant versus WT might display an atypical occurrence of uORFs. For the 70 genes with TE_4G_/TE_WT _values ≤ 0.71 whose occurrences of uORFs were tabulated by Lawless et al [16], there is an average of 0.43 ± 0.17 uORFs per transcript. For the 47 genes with TE_4G_/TE_WT _≥1.4, the corresponding average is 0.51 ± 0.26 uORFs per transcript. Neither of these frequencies differs significantly from the average uORF occurrence of 0.36 ± 0.02 uORFs per transcript tabulated for 4149 genes by Lawless et al. Thus, we found no indication that the presence or absence of uORFs is a critical determinant of the effect of eIF4G on the translational efficiency of eIF4G-responsive mRNAs.

In animals, translational control of specific mRNAs frequently involves trans-acting factors that bind to specific recognition elements in the 3'UTR and target eIF4F assembly at the cap structure [18]. Accordingly, we examined whether the 3'UTR length differs significantly between the two sets of genes identified above. As shown in Figure 7, the 3'UTR length appears to be slightly smaller for the group of genes with TE_4G_/TE_WT _≤ 0.71 versus that with TE_4G_/TE_WT _≥1.4 (P = 0.06); however, neither group displays a mean 3'UTR length that is significantly different from that of all genes. Hence, it seems unlikely that 3'UTR length is an important parameter in determining the dependence of translational efficiency on eIF4G.

Finally, we examined 10 mRNAs reported to have an A-rich IRES (*YMR181C, GPR1, BOI1, FLO8, NCE102, MSN1, GIC1, TPK2, HMS2, MTC7*) and also the IRES-containing mRNA *URE2*, to determine whether the translational efficiencies of these mRNAs might be increased or decreased on depletion of eIF4G. We observed no significant deviation from unity in the TE_4G_/TE_WT _ratios of the 10 genes with A-rich IRESs: 1.1 ± 0.06 (*YMR181C*), 1.00 ± 0.20 (*GPR1/YDL035C*), 1.4 ± 0.53 (*BOI1/YBL085W*), 1.1 ± 0.2 (*FLO8/YER109C*), 0.92 ± 0.10 (*NCE102/YPR149W*), 1.2 ± 0.11 (*MSN1/YOL116W*), 1.0 ± 0.13 (*GIC1/YHR061C*), 1.00 ± 0.16 (*TPK2/YPL203W*), 0.94 ± 0.38 (*HMS2/YJR147W*). The same holds true for the *URE2*/*YNL229C *mRNA, exhibiting a TE_4G_/TE_WT _ratio of 1.27 + 0.20 (P-value 0.13). These findings provide no indication that eIF4G is a critical factor for the function of these IRES elements, although it is unknown whether any of the IRESs are functional under the nonpermissive growth conditions of our experiments.

### Most genes with short ORFs require eIF4G to achieve their characteristic, higher than average translational efficiencies

Because we examined polysomal RNAs present in "heavy polysomes" (containing 4 or more 80S ribosomes), genes whose transcripts contain, on average, less then 4 translating ribosomes were likely underrepresented in this analysis. Of particular concern are those genes with relatively long coding regions whose mRNAs have an average of 3 or fewer translating ribosomes in WT cells, i.e., with low ribosome densities, because such inefficiently translated mRNAs might be particularly dependent on eIF4G. To address this possibility, we extended our microarray analysis to include RNA isolated from the "light polysome" (LP) fractions obtained from the same gradients that yielded the HP fractions analyzed above (see Additional file 3). The mean TE for each gene was calculated as the ratio of LP/T RNA intensities in all three projects, as above for HP RNA. We then cross-referenced the resulting TE values with a database listing the ribosome densities of 2,218 yeast genes described by Arava et al. [14], focusing on a group of 564 genes whose mRNAs in that study displayed peak occupancies of only 1-3 ribosomes per mRNA, and thus should occur primarily in the LP fractions of our study, of which 512 were interrogated on our microarrays. A subset of 133 genes from this group contain relatively long coding sequences (mean length of ~2000 nt) and exhibit average ribosome densities of ≤ 0.25 ribosomes per 100 nt-well below the genome-average density of 0.64 [14]. The mean TE_WT _calculated for these genes from our LP data, 0.81 + 0.03, is significantly below the genome average TE_WT _value of 1.100 ± 0.006 derived from the HP data for all 5869 ORFs, indicating that these genes exhibit an atypically low proportion of mRNA associated with ribosomes in addition to a low ribosome density. Consistent with our findings on mRNAs in HP fractions, the majority of these poorly translated mRNAs in the LP fractions exhibit higher TE values in the eIF4G mutant versus WT cells (Additional file 3, sheet "133 genes"). Thus, it appears that eIF4G is not a critical rate-limiting factor for this group of very inefficient mRNAs.

We also examined a subset of 245 genes from the group of 512 mentioned above, which exhibit peak occupancies of only 1-3 ribosomes per mRNA simply because they have short ORF lengths (< 625 nt), as their mean ribosome density (0.70) actually exceeds the genome-average of 0.64 [14]. Interestingly, these genes have a mean TE_WT _value of 1.96 ± 0.05 (in the LP dataset), that is substantially higher than the genome-average TE_WT _value (1.100 ± 0.006) and most of these genes have significantly lower TE values in the eIF4G mutant versus WT (mean TE_4G _of 1.69 ± 0.03) (Additional file 3, sheet "245 genes").

Having identified a group of efficiently translated mRNAs with a marked dependence on eIF4G that contain atypically short coding sequences, we examined the behavior of all genes with short ORFs (< 625 nt) in both the LP and HP data sets. As illustrated in the log-log plots of Figure S2 (Additional file 2), ~90% of these genes exhibit TE values greater than unity in WT cells (points above x-axis), compared to only 55% for genes of all ORF lengths (Figure 4). This disparity reflects the broader phenomenon that TE_WT _values are inversely related to ORF length, as revealed in the scatterplot of TE_WT _values versus ORF length for the entire HP dataset (Figure S3A in Additional file 2). This relationship is not unexpected, as it was noted previously that ribosome densities on mRNAs [14] and protein expression levels [19] are inversely related to ORF length in yeast. Interestingly, the majority of the short-ORF genes exhibit reductions in TE values in the eIF4G mutant of ~10% on average (Figure S2A-B in Additional file 2), similar to the average reduction in TE mentioned above for all genes with TE_WT _values above unity (Figure 4). The reduction in TE evoked by depletion of eIF4G for small-ORF genes is also obvious in the scatterplots of Figure S3 (Additional file 2), as dampening TE values for the shortest ORF lengths in the eIF4G mutant is observed. Thus, genes with short ORFs tend to be translated more efficiently in WT cells and to be dependent on eIF4G for their maximum efficiency.

It is noteworthy that the two sets of ~100 genes we identified above displaying the greatest changes in TE values on depletion of eIF4G differ dramatically in average ORF length. The group exhibiting the greatest reductions in translation efficiency (TE_4G_/TE_WT _≤ 0.71) has a mean ORF length below the genome average by nearly a factor of two (P < 0.0001), while genes showing the greatest increases in efficiency (TE_4G_/TE_WT _≥ 1.4) have a mean ORF length 70% larger than average (P < 0.0001) (Figure 7). These findings suggest that ORF length, in addition to 5'UTR length, determines the influence of eIF4G on translational efficiency. Below, we propose a molecular explanation for this finding, based on the known relationship between transcript length and the stability of eIF4F-cap interaction [20].

Considering the strong correlation between ORF length and effect of eIF4G depletion on translational efficiency shown in Figure 7, it seems possible that the enrichment of cellular functions associated with the gene sets exhibiting TE_4G_/TE_WT _≤ 0.71 or TE_4G_/TE_WT _≥1.4 described above (Figure 6) could at least partially reflect a preponderance of genes with unusually small or large ORF lengths in those functional categories.

## Discussion

In this study, we have examined the genome-wide consequences for translational efficiency of simultaneously eliminating eIF4G2 and depleting eIF4G1 from yeast cells. The conditional depletion of eIF4G1 achieved using a degron-tagged version of this protein was highly effective and reduced the polysome content and rate of translation to only 20-30% of WT levels, indicating a substantial reduction in the rate of translation initiation. We used genome expression microarrays to measure the abundance of each mRNA in heavy polysomes (with 4 or more translating ribosomes) relative to its level in total mRNA to calculate translational efficiencies of 5868 different genes. The results indicated that the overwhelming majority of mRNAs experienced only a moderate change in translational efficiency on eIF4G depletion. Less than 2% of the genes showed a statistically significant decrease in TE in the mutant by a factor of 1.4 of more, and the genes in this group that were affected the most displayed reductions of a factor of ~2.5 or less. While the actual percentage of genes affected to this extent is probably higher, only ~10% of genes exhibited decreases in TE of this magnitude for each biological replicate, which likely represents the upper size limit for this category. Thus, we did not detect even a small group of mRNAs that are dramatically dependent on eIF4G for translation in vivo.

We made the unexpected observation, however, that depletion of eIF4G narrows the range of translational efficiencies for a large fraction of mRNAs, decreasing the number with efficiencies that are substantially higher or lower than the genome-average TE. This trend is well illustrated in the log-log plots of mean TE values in WT versus mutant cells (Figure 4), and also by the fact that depleting eIF4G reduced (by several fold) the numbers of mRNAs with TE values either 1.5-fold higher, or 1.5-fold lower, than unity (Table 1). Furthermore, the bulk of mRNAs with TE values ≥ 1.5 in WT cells are, at least to some extent, dependent on eIF4G for their higher than average TE values (Table 1). This dependence is consistent with a significant role for eIF4G in stimulating one or more steps of initiation for the most efficiently translated mRNAs in the cell, presumably the activation of mRNA for recruitment of the 43S PIC, scanning the 5'UTR, or start codon recognition. Unexpectedly, we found that many mRNAs with lower than average TE values in WT cells exhibit an increased translational efficiency on eIF4G depletion. It is conceivable that eIF4G directly impairs the translation of these latter mRNAs. However, we favor an indirect mechanism involving competition among all mRNAs for limiting initiation factors or PICs, coupled with the role of eIF4G in stimulating efficiently translated mRNAs at the expense of those with lower than average efficiencies. In the absence of eIF4G, this competitive edge would be eliminated for the first group and thereby enable the second group of mRNAs to compete better for limiting factors/PICs.

The small group of ~100 genes we identified that are most dependent on eIF4G for their higher than average TEs in WT cells contain a mean 5'UTR length that is slightly below the genome-average for all mRNAs (Figure 7), a feature that should facilitate efficient scanning and AUG recognition. This was surprising because we expected to find that the mRNAs most dependent on eIF4G would have long or highly structured 5'UTRs, requiring the eIF4G·eIF4A complex for unwinding secondary structure to promote 43S attachment or scanning. In fact, the ~100 genes we identified whose translation is stimulated the most by eliminating eIF4G contain a mean 5'UTR length substantially larger than the genome average (Figure 7). The fact that these latter mRNAs display a lower than average TE in WT cells and benefit from the absence of eIF4G seems to indicate that they function inefficiently at steps of initiation not significantly enhanced by eIF4G. Given their long 5' UTR lengths, it seems likely that scanning to the start codon is relatively inefficient for these mRNAs. If so, then the fact that depleting eIF4G does not exacerbate this deficiency suggests that factors besides eIF4G are more critically required for efficient scanning through long 5'UTRs in yeast.

This last suggestion is consistent with our finding that none of the 17 mRNAs predicted by the Randfold program to contain the most stable secondary structures among yeast 5' UTRs [16] displayed a significant reduction in TE on eIF4G depletion--in fact, four such mRNAs appear to be translated more efficiently on eIF4G depletion. Thus, other initiation factors besides eIF4G might also be more critically involved in removing secondary structures in advance of the scanning PIC. This view is supported by the fact that in a mammalian reconstituted system, eIF4G, eIF4A and eIF4B are sufficient for 43S attachment and scanning on β-globin mRNA, which harbors a relatively unstructured 5'UTR, whereas the DExH-box protein DHX29 is required for initiation complex assembly on mRNAs containing more structured 5'UTRs [7]. Similarly, there is evidence that yeast DEAD-box protein Ded1 contributes more than eIF4A does to the processivity of scanning in vivo [6]. These findings are in agreement with the possibility that the eIF4E/eIF4G/eIF4A complex (eIF4F) is more critical for 43S PIC attachment near the 5' end of the mRNA than for subsequent scanning to the start codon.

Thus, our results are consistent with the model that 43S attachment is a rate-limiting step for a large proportion of mRNAs with higher than average TEs, and that this step is stimulated by eIF4G, particularly for the ~100 genes we identified with the greatest dependence on eIF4G that contain relatively short 5'UTRs. By contrast, scanning or AUG recognition would be rate-limiting for mRNAs with longer than average 5'UTRs whose translation is enhanced by depletion of eIF4G, because these steps are not critically dependent on eIF4G. The fact that eliminating eIF4G mitigates the lower than average translational efficiencies of this second group of mRNAs can be explained by proposing that the negative effect of depleting eIF4G on 43S attachment is outweighed by their enhanced ability to compete with other mRNAs for limiting factors that promote scanning or AUG recognition.

Fulfilling this last stipulation of our model would be facilitated if the inefficient mRNAs with long 5'UTRs are relatively ineffective at exploiting eIF4G function in 43S attachment. That is, if eIF4G contributes relatively less to 43S attachment by these inefficient mRNAs in WT cells, then depleting eIF4G would produce relatively smaller reductions in their translation rate. One reason for thinking that this condition holds is our finding that this group of mRNAs also displays unusually long coding sequences, whereas the mRNAs we identified with the greatest dependence on eIF4G exhibit smaller than average ORF lengths. Recent findings by Jacobson et al [20] indicate that shorter yeast mRNAs produce more stable eIF4F-cap interactions than do longer mRNAs, which is fully dependent on an extended poly(A) tail and PABP. Presumably, shorter mRNAs more efficiently assemble a closed-loop mRNP via PABP-eIF4G interaction, which stabilizes eIF4F binding to mRNA [3,21]. In fact, the possibility of less efficient 5'-3' interaction for larger mRNAs was advanced previously as one explanation for the inverse correlation between ribosome density and ORF length [14], which we confirmed here using TE values (Figure S3A in Additional file 2). Hence, we suggest that longer mRNAs are affected less than shorter mRNAs by the elimination of eIF4G because the eIF4F-cap interaction is inherently less stable for longer transcripts and, hence, less efficacious in promoting 43S recruitment when eIF4G is present. The fact that depleting eIF4G diminishes, but does not eliminate the correlation between TE and ORF length (Figure S3B in Additional file 2) indicates that reduced eIF4G-PABP interaction is not the only factor limiting the translation of mRNAs with longer ORFs, and limited processivity of elongating ribosomes or less efficient termination have been suggested as other possibilities [14].

We showed previously that depletion of eIF4G did not lower the amounts of native 48S complexes containing the *RPL41A *or *MFA2 *mRNAs [8], both very short transcripts, which is ostensibly at odds with the idea that eIF4G has an important function in 43S attachment to mRNA. Examining the results we obtained for these mRNAs in the LP dataset (from small polysomes) reveals that they both exhibit mean TE_4G _values ~90% of their TE_WT _values (Additional file 3). Thus, even if we assume that these two mRNAs require eIF4G only at the step of 43S attachment to achieve their maximum translation rates, it would have been very difficult to detect a 10% decrease in the levels of their free 48S complexes with the techniques employed in the previous study [8]. It remains to be determined what features in mRNA, besides a short 5'UTR and short ORF length, are responsible for the more pronounced requirement for eIF4G displayed by the small fraction of yeast mRNAs identified here.

Considering that eIF4G is essential in yeast, and also noting its role as a protein bridge linking the eIF4E-mRNA-PABP mRNP to components of the 43S complex (eIF5 and eIF1) [22], it is surprising that a significant amount of translation still proceeds in the absence of this factor. Based on our microarray data, it appears that eIF4G is dispensable for the translation of most, if not all mRNAs in vivo, indicating that it is rate-enhancing rather than essential in budding yeast. This stands in contrast to the critical requirement for the eIF3 complex, which is required for nearly all translation in yeast, and is crucial for attachment of native 43S complexes to mRNAs (*RPL41A *and *MFA2) *that can assemble 48S PICs in cells depleted of eIF4G [8]. Of course, we cannot exclude the possibility that a compensatory initiation pathway comes into play during the 8 h of incubation in the non-permissive conditions used to thoroughly deplete eIF4G. It is also impossible to eliminate the possibility that a very small fraction of the WT amount of eIF4G, below the detection limit of our Western analysis, is sufficient to catalyze the residual protein synthesis that occurs in the depleted cells. This seems unlikely, however, because the eIF4G level in WT cells is already lower than those of nearly all other initiation factors [23].

On the other hand, the 3 to 4-fold reduction in the rate of translation, and the narrowed range of translational efficiencies evoked by depletion of eIF4G, could have serious consequences for a subset of dosage-sensitive proteins with essential functions in the cell. Moreover, cell division could be blocked under these conditions by regulatory mechanisms that respond to a drop in the rate of synthesis of a key cell cycle controlling factor, eg., the G1 cyclin Cln3 [24-26]. Considering that cell division is not blocked by a decrease in the overall translation rate of ~70% occurring in response to hyperosmotic stress [27,28], eIF4G depletion might evoke a comparatively greater reduction in translation of a key protein(s) required for cell division than occurs during osmotic stress.

Given that depletion of eIF4G reduces the translation rate by 3 to 4-fold, it is surprising that the average TE calculated for all 5868 genes decreased only a small amount, from 1.100 ± 0.006 in WT cells to 1.05 ± 0.004 in the mutant. Of course, many genes translated with higher than average efficiencies in WT exhibit much larger reductions in TE values on depletion of eIF4G, but this effect was counterbalanced by increased translation of many genes with lower than average TE_WT _values. As noted above, the fact that microarray results are normalized to give each array the same average signal intensity will dampen the reduction in polysomal mRNA abundance in the eIF4G mutant, and the amounts of total mRNA might also decline on eIF4G depletion, which would offset the effect of decreased polysomal mRNA on the calculated TE values. It is also conceivable that eIF4G depletion triggers a signal transduction response that decreases the rate of elongation, counteracting the effect of reduced initiation on polysome size. For example, oxidative stress reduces the rates of both initiation and elongation in yeast [29].

Because we examined cells lacking eIF4G2 and depleted of eIF4G1, it could be argued that the changes in translational efficiencies we observed result primarily from the absence of only eIF4G1 or eIF4G2 rather than the elimination of both eIF4G isoforms. This is unlikely in view of recent findings by Clarkson et al on mutant strains expressing only eIF4G1 or eIF4G2 and engineered to express each isoform at a level equivalent to the combination of both isoforms in WT. These strains displayed almost no changes in translational efficiency genome-wide [30], providing strong evidence against the possibility that eIF4G1 or eIF4G2 is specifically required to support the translation of particular mRNAs. In this same study, two groups of protein-coding genes (of ~150 each) displayed a significant change in translational efficiency on deletion of only *TIF4631*, encoding the major isoform (eIF4G1), which reduced the growth rate and polysome content relative to the isogenic WT strain. Only 10% of the genes with significantly repressed translational efficiencies in *tif4631Δ *cells thus identified by Clarkson et al belong to the group of ~100 genes we identified here with mean TE_4G_/TE_WT _ratios of ≤ 0.71. However, the group of translationally repressed genes in the Clarkson et al study displayed an average TE_4G_/TE_WT _ratio in our experiments (0.89 ± 0.01) that is significantly below the genome-average TE_4G_/TE_WT _ratio (1.05 ± 0.004) and also the average TE_4G_/TE_WT _ratio determined in our experiments for the group of translationally enhanced genes identified by Clarkson et al (1.17 ± 0.02). Thus, the translational efficiencies of at least a subset of genes are affected similarly by the absence of eIF4G1 alone and the elimination of both eIF4G1 and eIF4G2 simultaneously. This is consistent with the conclusion that eIF4G1 and eIF4G2 perform essentially identical functions [30].

A recent analysis of the consequences of depleting eIF4GI and eIF4GII with siRNAs in cultured mammalian cells [31] reached certain conclusions congruent, and others that seem to differ, from our findings. It was found that depleting both eIF4GI and eIF4GII reduced overall translation by only ~20%, whereas depleting two eIF3 subunits provoked a stronger (~50%) reduction, consistent with the greater requirement for eIF3 versus eIF4G we observed in yeast [8]. eIF4GI depletion reduced the translational efficiencies of a subset of mammalian mRNAs, including a group whose products function in mitochondrial regulation, bioenergetics, and cell proliferation. In accordance with our observations, there was no significant correlation between the presence of long or structured 5'UTRs and the degree of eIF4GI-dependence. This is consistent with the aforementioned suggestion that eIF4GI is more important for 43S attachment than for subsequent scanning through the 5'UTR. At odds with our results, however, the eIF4GI-dependent class of mRNAs appeared to be somewhat enriched in those containing uORFs, and the presence of an uORF was shown to increase the eIF4GI dependence on translation. One possibility is that the majority of uORF-containing mRNAs in yeast do not support appreciable reinitiation in WT cells, as this process has strict requirements for uORF length and cis-acting sequences surrounding the stop codon [32,33]. In this event, eliminating the potential role of eIF4G in stimulating reinitiation would be difficult to detect on a genome-wide basis in yeast.

## Conclusions

Our results indicate that eliminating both isoforms of eIF4G from yeast cells elicits a substantial reduction in the rate of translation initiation that is severe enough to block cell division, but does not evoke dramatic changes in the relative translational efficiencies of the majority of mRNAs. Rather, we observed a large-scale narrowing of translational efficiencies, including mRNAs with higher or lower than average efficiencies, which is expected to disturb the stoichiometry of protein components comprising many cellular pathways and structures. Our finding that mRNAs with the greatest dependence on eIF4G are relatively well-translated, do not contain long or highly structured 5'UTR, and also have short coding sequences, is consistent with the idea that eIF4F is most critically required to enhance 43S attachment to the mRNA 5' end rather than for scanning through long, structured 5' UTRs.

## Methods

### Yeast strains

The following yeast strains employed in this study were described previously [8]: YAJ3 (*MAT***a ***trp1Δ leu2-3,112 ura3-52 gcn2Δ *::*hisG P_GAL1 _-myc-UBR1 *::*TRP1 *::*ubr1*, pRS316 <*URA3*>), YAJ41(*MAT***a ***trp1Δ leu2-3,112 ura3-52 gcn2 *::*hisG P_GAL1_-myc UBR1 *::*TRP1 *::*ubr1 tif4632Δ *::*kanMX6 P_CUP1 _-UBI-R-DHFR ^ts ^-HA-tif4631-td *::*URA3 *::*tif4631*), and YAJ34 (*MAT***a ***trp1Δ leu2-3,112 ura3-52 gcn2Δ *::*hisG P_GAL1 _-myc-UBR1 *::*TRP1 *::*ubr1 P_CUP1 _-UBI-R-DHFR^ts ^-HA-prt1-td::URA3::prt1 P_CUP1 _-UBI-R-HA-tif32-td::URA3::tif32*).

### Yeast cell culture, sucrose gradient centrifugation, and RNA isolation

WT strain YAJ3, eIF4G1 degron mutant YAJ41, and eIF3 degron mutant YAJ34 were grown in liquid synthetic complete (SC) medium containing 2% raffinose as carbon source and 0.1 mM copper sulfate at 25 C (SC_Raf _+ Cu^2+^, 25°C; permissive conditions) to an optical density (A_600_) of 0.15 to 0.6. After addition of galactose (2%), cells were incubated for an additional 30 min at 25°C followed by growth in SC containing 2% raffinose, 2% galactose, and 1 mM bathocuproinedisulfonic acid (BCS) at 36°C (SC_Raf/Gal _+ BCS, 36°C; nonpermissive conditions) for up to 8 h. Cycloheximide was added to a final concentration of 0.1 mg/mL, and the culture was chilled on ice for 10 min. Cells were pelleted by centrifugation, resuspended in breaking buffer [20 mM Tris-HCl, pH 8.0, 50 mM KCl, 10 mM MgCl_2_, 2 mM dithiothreitol, 1% Triton X-100, 0.1 mg/mL cycloheximide, 0.5 mg/mL Heparin, 10 mM NaF, 0.5 mM AEBSF, 5 μg/mL leupeptin, Complete protease inhibitor cocktail tablets (EDTA-free, Roche Diagnostics)], and broken by vortexing with glass beads. Polysomes were separated by loading whole cell extracts (WCEs) onto 4.5-45% sucrose gradients and centrifuged in a SW41Ti rotor (Beckman) at 39,000 rpm for 2.5 h at 4°C as described previously [8]. Total RNA was isolated from the input WCE, or from pooled gradient fractions containing 80S monosomes, polysomes with 2-3 ribosomes (light polysomes, LP), or polysomes with 4 or more ribosomes (heavy polysomes, HP) using TRIZOL reagent (Invitrogen) according to the manufacturer's suggested protocol. Heparin was eliminated by precipitating the RNA with LiCl to a final concentration of 1.9 M followed by centrifugation in a microcentrifuge at 13,200 at 4°C. The pellet was washed with ethanol and dissolved in RNAse-free water. After addition of sodium acetate (pH 5.5) to a final concentration of 0.3 M, RNA was again ethanol precipitated, pelleted, and redissolved in RNAse-free water.

For the Western blot analysis in Figure 1A, WCEs were prepared as described above, resolved by 4-20% SDS-PAGE, and subjected to immunoblotting using rabbit polyclonal anti-eIF4G1 antibodies (a kind gift from John McCarthy) or mouse monoclonal anti-Pab1 antibodies (1G1, a kind gift from Maurice Swanson).

### *In vivo *[^35 ^S]-methionine incorporation

Yeast strains were grown to A_600 _of 0.25 to 0.6 under permissive conditions and further incubated for 8 h under nonpermissive conditions, as described above. One hour before labeling, cells were washed and resuspended in [SC_Raf/Gal _+BCS] lacking methionine. At the zero time point, unlabeled methionine was added at 50 μM and [^35 ^S]-methionine (7.9 mCi/ml, 293.0 MBq/ml, NEN Life Science Products) was added at 5 μCi/ml to each culture. At 15-min intervals, the A_600 _of the cultures was determined, and 1-ml aliquots were mixed with 0.2 ml of cold 50% trichloroacetic acid (TCA), incubated on ice for 10 min, boiled for 20 min and filtered through Whatman GF/C filters. Filters were washed with 5% cold TCA, 95% ethanol, dried, and the radioactivity quantified by liquid scintillation.

### Microarray analysis

Total RNA samples from the WCE or RNA samples from heavy polysomes were isolated as described above and sent to Roche-NimbleGen (Reykjavik, Iceland) for complete expression array services, including cDNA synthesis, Cy3-cDNA labeling, and hybridization of microarrays according to their standard protocols. Briefly, cDNA was synthesized from 10 μg of RNA, labeled with Cy3, and hybridized to three replicate NimbleGen *S. cerevisiae *1-plex 385K arrays (Cat # A4345001-00-01; NimbleGen) for each RNA sample. Following washing and scanning of the arrays, data was extracted from the scanned image and analyzed for normalized gene expression summary values (CALLS) by quantile normalization and the Robust Multi-array Average (RMA) algorithm [34] using the NimbleScan software (NimbleGen). ArrayStar 3.0 software (DNASTAR; Madison, WI) was used to analyze the expression data provided by NimbleGen. Mean TE_4G _and TE_WT _values were calculated for each gene from all nine microarray measurements of HP or T mRNA intensities obtained in the three biological replicates (projects I-III) to obtain the log-log plot in Figure 4. To calculate mean TE_4G_/TE_WT _ratios for the purpose of assigning standard errors to the values, the ratios were calculated separately for each project from the mean TE_4G _and mean TE_WT _values calculated from the three technical replicates for that project, and the resulting TE_4G_/TE_WT _ratios for each project were averaged. The three mean TE_4G _and mean TE_WT _values determined in this way from projects I-III were also used to conduct two-tailed Student's t-tests of the significance of differences between mean TE_4G _and mean TE_WT _values for individual genes.

### Accession number

The microarray data discussed in this publication have been deposited in NCBI's Gene Expression Omnibus and are accessible through GEO Series accession number GSE25721 http://www.ncbi.nlm.nih.gov/geo/query/acc.cgi?acc=GSE25721.

### Real-time quantitative RT-PCR (qRT-PCR) analysis of polysomal mRNA distributions

RNA samples from the WCE or gradient fractions containing HP, LP, or 80S monosomes were isolated as previously described. The level of mRNA for each gene of interest (GOI) relative to the amount of 18S rRNA was quantified by qRT-PCR analysis. Briefly, cDNA was synthesized from 1 μg of RNA using SuperScript™ III First-Strand Synthesis SuperMix (Invitrogen) according to the vendor's recommended protocol. The synthesized first-strand cDNA was diluted 1:10, and 2 μl of the diluted cDNA was used for subsequent real-time PCR amplification using the Stratagene MX3000P and Brilliant II SYBR^® ^Green QPCR Master Mix (Stratagene) according to the vendor's instructions. The primers used in qRT-PCR analysis for the mRNAs analyzed in Figure 2 are listed in Table S2 (see Additional file 2). The real-time PCR reactions were carried out in triplicate for each cDNA sample to obtain average Ct values. The amount of mRNA in a set of gradient fractions containing HP, LP or 80S species relative to its level in total RNA was determined by first calculating 2^-ΔΔCt^, where ΔΔCt = Ct(fraction)_norm _- Ct(total)_norm_, Ct(fraction)_norm_= Ct(fraction)_GOI _- Ct(fraction)_18S_, and Ct(total)_norm _= Ct(total)_GOI _- Ct(total)_18S_. Ct(fraction)_GOI _and Ct(total)_GOI _are the Ct values determined for the gene of interest in the appropriate gradient fractions or total RNA, respectively; Ct(fraction)_18S _and Ct(total)_18S _are the corresponding values for 18S rRNA. The resulting 2^-ΔΔCt ^values were then multiplied by a factor representing the proportion of the total A_280 _units in the gradient found in the appropriate fractions (HP, LP or 80S, respectively). These factors were calculated by integrating the A_280 _values from the polysome tracings for the appropriate fractions from multiple independent experiments on WT and mutant (4G) extracts, yielding the following average values: HP_WT _= 0.308, HP_4G _= 0.114, LP_WT _= 0.276, LP_4G _= 0.149; 80S_WT _= 0.416; 80S_4G _= 0.738.

The TE_4G_/TE_WT _values plotted in Figure S1 (Additional file 2) were calculated as (2^-[ΔΔCt(HP-T)] ^_4G_/2^-[ΔΔCt(HP-T)] ^_WT_)(0.114/0.308), where 0.114 and 0.308 are the fractions of 18S rRNA present in heavy polysomes in the mutant and WT cells, respectively, ΔΔCt(HP-T) = ΔCt(HP)_norm _-ΔCt(T)_norm_, ΔCt(HP)_norm _= Ct(HP_GOI_)-Ct(HP_18S_), and ΔCt(T)_norm _= Ct(T_GOI_)-Ct(T_18S_). The primers employed for qRT-PCR analysis of the mRNAs for these genes are listed in Table S3 (see Additional file 2).

## Authors' contributions

EHP participated in the design of the study, carried out the biochemical analyses of protein synthesis and prepared the polysomal and total RNAs for microarray analysis. FZ carried out the real-time PCR analyses. JW conducted the statistical analysis of the enrichment for specific cellular functions, and for essentiality, in the gene sets described in Figure 6. PS participated in the design of the study and helped to draft the manuscript. AGH participated in the design and coordination of the study, performed statistical analyses, and helped to draft the manuscript. All authors read and approved the final manuscript.

## Supplementary Material

Additional file 1 **Excel file containing results of microarray analysis of translational efficiencies using RNA from "heavy polysomes" (HP) and total RNA isolated from WT and eIF4G-depleted cells**. Spreadsheet 1 "microarray of 5869 ORFs" contains the data from microarray analysis of 5869 ORFs using HP and total RNA samples from WT and eIF4G mutant strains, from projects (biological replicates) I-III. Columns A-C contain the Nimblegen sequence identification number, SGD ORF name, and functional annotation, respectively. Columns D-F contain the log_2 _of signal intensity for the HP RNA sample from the eIF4G mutant in project I (log4G-HP_I), the cognate value for total RNA (log4G-T_I), and the corresponding TE value (TE 4G_I) calculated as the ratio of these two intensities (HP/T), respectively. Columns G-I contain the corresponding data found in D-F for the HP and T RNA samples but from the WT strain in project I. Columns J-L and M-O contain the corresponding data found in D-F and G-I but from project II, and columns P-R and S-U contain the cognate data from project III. Columns V and W contain the mean TE_4G _and mean TE_WT _values, respectively, obtained by averaging the TE values from projects I-III. The log_2 _of the values in columns V and W were plotted in Figure 4. Columns X, Y, and Z contain the ratios of the TE_4G _to TE_WT _values from projects I, II, and III, respectively, contained in columns F and I, L and O, and R and U, respectively. Column AA contains the average of columns X-Z, AB contains the S.E.M. calculated from the data in X-Z, and column AC contains the results of a two-tailed Students t-test conducted to determine if the set of TE_4G _values in columns F, L, and R are significantly different from the set of TE_WT _values in columns I, O, and U. Spreadsheet 2 "t-test calcs" contains these last calculations. Spreadsheet 3 "(p < 0.1, TEg 0.71 TEwt)" contains the 94 genes with mean TE_4G_/TE_WT _values (in column AA) ≤ 0.71, and also includes in columns AD-AI the lengths and MFEs of 5' UTRs, and numbers of uORFs, all from [16], and 3' UTR and ORF lengths calculated from Nagalakshmi et al [35]. Spreadsheet 4 "p < 0.1, TEg 1.4 TEwt" contains the 99 genes with mean TE_4G_/TE_WT _values (in column AA) ≥ 1.4, with the identical column definitions described above for sheet 3. Spreadsheet 5 "Lawless UTRs" contains the data from Additional file 9 of Lawless et al [16]. Spreadsheet 6 "Lawless MFEs" contains the data from Additional file 8 of Lawless et al [16]. Click here for file

Additional file 2 **Comparison of translational efficiencies between WT and eIF4G degron mutant.** -Figure S1: Comparison of the ratios of TE values between the WT and eIF4G degron mutant determined by qRT-PCR and microarray analysis of selected mRNAs. -Figure S2: Comparison of translational efficiencies for all genes with ORF lengths < 625 nt between WT and eIF4G degron mutant cells. -Figure S3: Inverse correlation between translational efficiency and coding sequence length is dampened by eIF4G depletion, especially for genes with short ORFs. -Table S1: Variances from MA plots of microarray data. -Table S2: PCR Primers for measuring polysome distributions of selected mRNAs. -Table S3: Primers for qRT-PCR determination of TE4G/TEWT ratios and comparison of mean TE4G/TEWT ratios determined by microarray versus qRT-PCR analysis. Click here for file

Additional file 3 **Excel file containing results of microarray analysis of translational efficiencies using RNA from "light polysomes" (LP) and total RNA isolated from WT and eIF4G-depleted cells**. Spreadsheet 1 "Project averages LP over T_4G,WT" contains our data from microarray analysis of 5868 ORFs using LP and total RNA samples from WT and eIF4G mutant strains, from all three projects (I-III). Columns A-B contain the Nimblegen sequence identification number and SGD ORF name, respectively. Columns C-F contain the mean signal intensities, averaged over all three technical replicates and all three projects (9 microarrays in total) for the LP and total RNA samples from the eIF4G mutant (C-D) and from WT (E-F), respectively. Columns G and H contain the mean TE_4G _and mean TE_WT _values, respectively, calculated as the ratio of column C to column D (column G), or the the ratio of column E to column F (column H). Column I is the ratio of column G to column H. Spreadsheet 2 "Arava et al, 1-3 ribos per mRNA" contains the list of 564 genes whose polysomal mRNAs contain predominately 1-3 ribosomes, from Arava et al [14]. Spreadsheet 3 "512 genes, 1-3 ribosomes" contains the same information as sheet 2 (columns A-H) for the subset of 512 genes from sheet 2 that were represented in our microarrays. Columns (I-K) contain the cognate information from columns G-I of sheet 1. Spreadsheet 4 "133 genes, 1-3 ribosomes" is analogous to sheet 3 for the subset of 133 genes from sheet 2 with ribosome densities of ≤ 0.25 ribosome per 100 nt. Spreadsheet 5 "245 genes, 1-3 ribosomes" is analogous to sheet 3 for the subset of 245 genes from sheet 2 with ORF lengths < 625 nt. Click here for file
