# Supplementary material for: Depletion of eIF4G from yeast cells narrows the range of translational efficiencies genome-wide
Source: BMC Genomics. 2011 Jan 26;12:68. doi: 10.1186/1471-2164-12-68 (PMC3042410; doi:10.1186/1471-2164-12-68)
Supplement: Additional file 2 — Comparison of translational efficiencies between WT and eIF4G degron mutant. -Figure S1: Comparison of the ratios of TE values between the WT and eIF4G degron mutant determined by qRT-PCR and microarray analysis of selected mRNAs. -Figure S2: Comparison of translational efficiencies for all genes with ORF lengths < 625 nt between WT and eIF4G degron mutant cells. -Figure S3: Inverse correlation between translational efficiency and coding sequence length is dampened by eIF4G depletion, especially for genes with short ORFs. -Table S1: Variances from MA plots of microarray data. -Table S2: PCR Primers for measuring polysome distributions of selected mRNAs. -Table S3: Primers for qRT-PCR determination of TE4G/TEWT ratios and comparison of mean TE4G/TEWT ratios determined by microarray versus qRT-PCR analysis. [file 1471-2164-12-68-S2.PDF]

Additional file 2 for:

Depletion of eIF4G from yeast cells narrows the range of translational efficiencies genome-wide

Eun-Hee Park[1], Fan Zhang[1], Jonas Warringer[2], Per Sunnerhagen[2], and Alan G. Hinnebusch [1†]

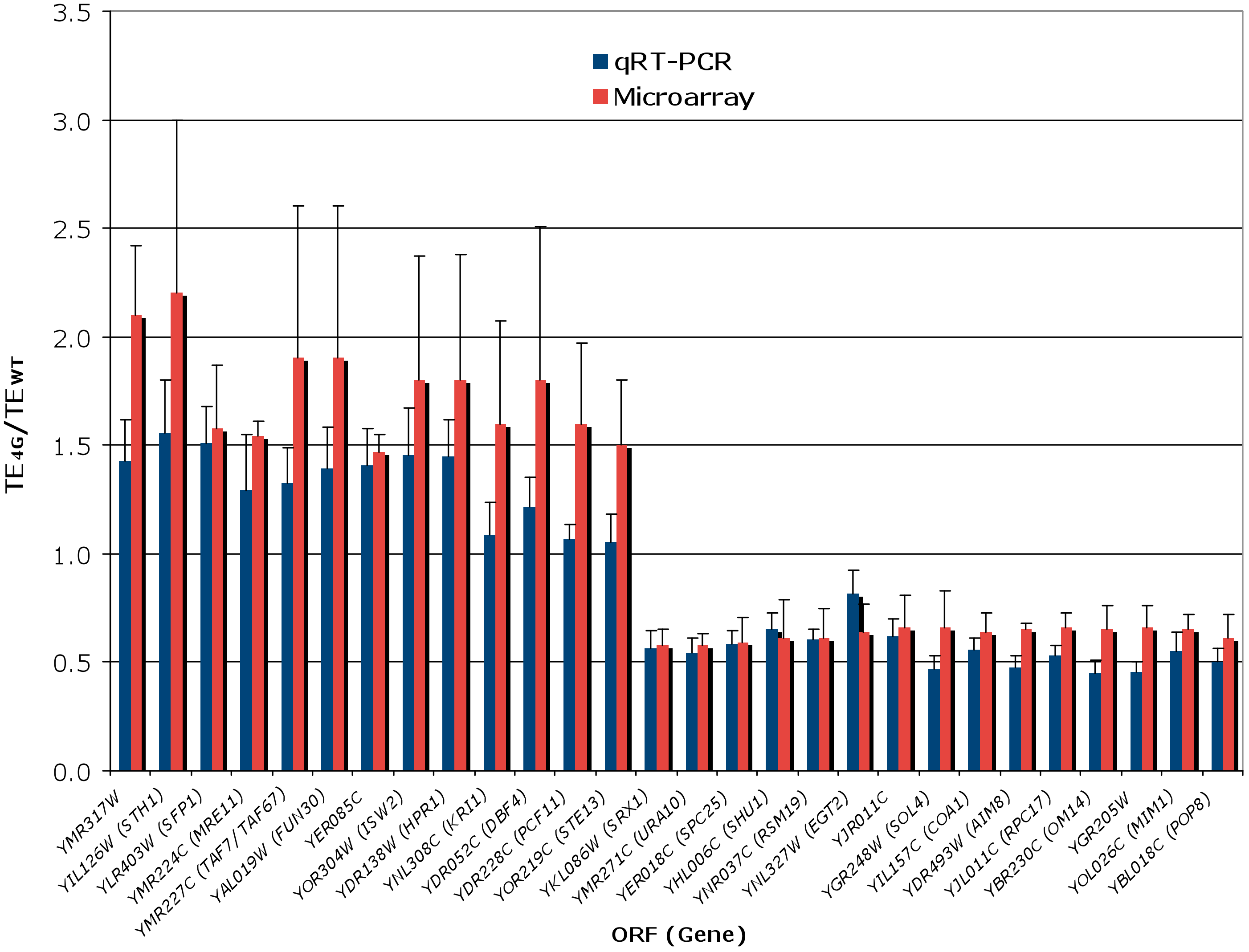

Figure S1. Comparison of the ratios of TE values between the WT and eIF4G degron mutant determined by qRT-PCR and microarray analysis of selected mRNAs. The mean TE_{4G}/TE_{WT} ratios (+/- S.E.M.) in projects I-III determined by qRT-PCR (dark blue) or microarray analysis (red) are plotted for the indicated genes, listed by their ORF and gene designations. For the genes with $TE_{4G}/TE_{WT} \leq 0.71$, the values measured by PCR analysis correspond closely to those calculated by microarray analysis. There appeared to be less agreement between the two measurements for genes with $TE_{4G}/TE_{WT} \geq 1.4$, although in view of

the higher variances for the microarray measurements, the two sets of results are not statistically different. Because the microarray measurements generally exceeded the values obtained by qRT-PCR analysis for this second class of mRNAs, such genes might exhibit smaller relative increases in translational efficiency in the mutant than are indicated by the microarray measurements. Table S3 lists the primers used for the qRT-PCR analysis and also the values plotted here in the histogram.

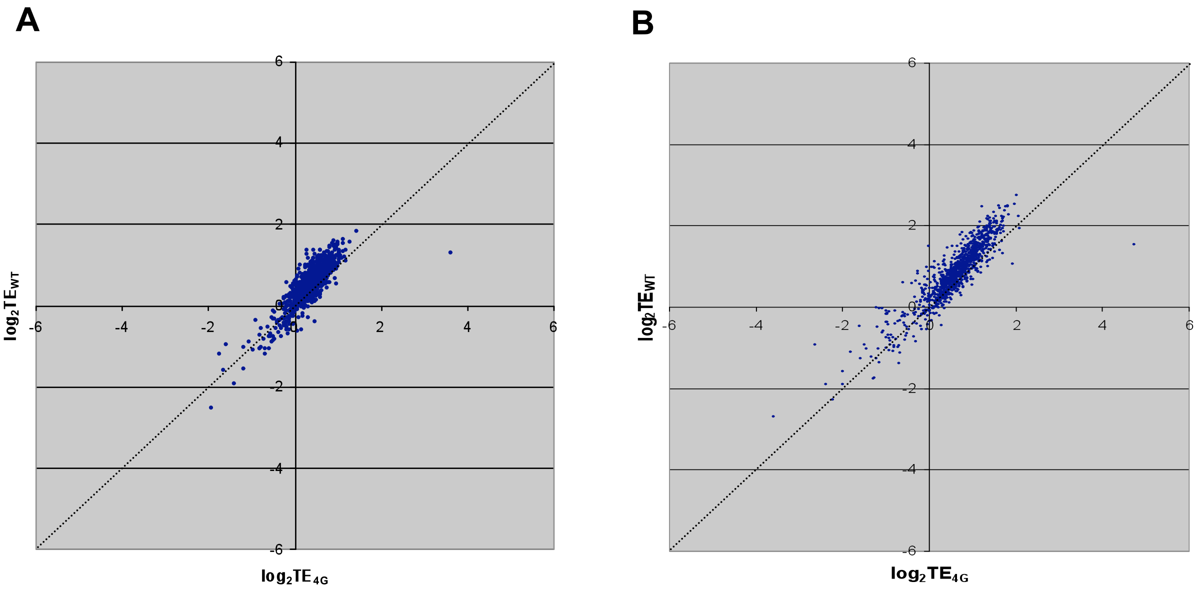

Figure S2. Comparison of translational efficiencies for all genes with ORF lengths <625nt between WT and eIF4G degron mutant cells. (A) $\log_2$(TE) values were determined from the mean TE values calculated using data from all three biological replicates obtained from the heavy (HP) dataset (Additional file 1) for the subset of genes with ORF lengths <625nt, using ORF lengths calculated from Table S3 of Nagalakshmi et al [1]. The dotted line is the theoretical regression line in the hypothetical situation where TE values for all genes are identical between mutant and WT cells. (**B**) Identical to (A) except that the light polysome (LP) dataset (Additional file 3) was employed.

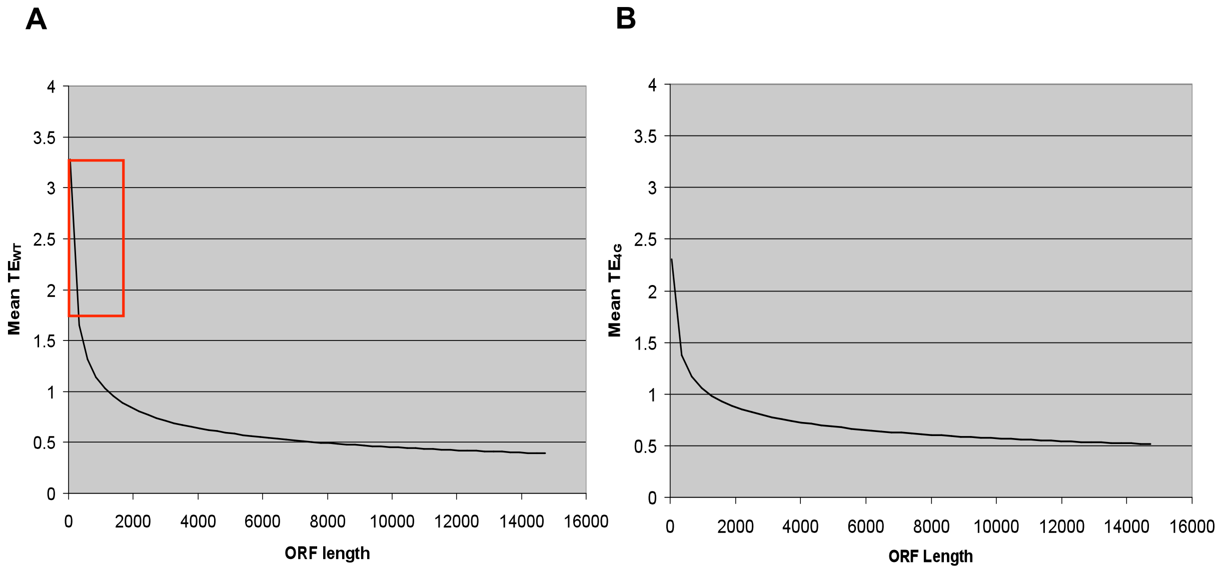

Figure S3. Inverse correlation between translational efficiency and coding sequence length is dampened by eIF4G depletion, especially for genes with short ORFs. (A) Scatterplots of mean translational efficiencies versus ORF length (in nucleotides) for all genes in WT cells, calculated using data from all three projects for the HP gradient fractions. **(B)** Same as in (A) except for the eIF4G degron mutant. The red box in (A) highlights the region of the plot where the inverse relationship between ORF length and TE is dampened in the plot shown in panel B for the eIF4G mutant.

Table S1. Variances from MA plots of microarray data[1]

Comparison	σ^2 of *M* values
Biological Replicates	
HP_{4G} vs. HP_{4G}	
1. I vs. II	0.087
2. I vs. III	0.034
3. II vs. III	0.09
	$\overline{X} = 0.07$
HP_{WT} vs. HP_{WT}	
4. I vs. II	0.17
5. I vs. III	0.26
6. II vs. III	0.13
	$\overline{X} = 0.19$
T_{4G} vs. T_{4G}	
7. I vs. II	0.065
8. I vs. III	0.044
9. II vs. III	0.081
	$\overline{X} = 0.063$
T_{WT} vs. T_{WT}	
10. I vs. II	0.051
11. I vs. III	0.059
12. II vs. III	0.044
	$\overline{X} = 0.051$
Mutant vs. WT comparisons	
HP_{4G} vs. HP_{WT}	
13. I vs. I	0.36
14. II vs. II	0.41
15. III vs. III	0.40
	$\overline{X} = 0.39$
T_{4G} vs. T_{WT}	
16. I vs. I	0.28
17. II vs. II	0.24
18. III vs. III	0.20
	$\overline{X} = 0.24$

Table S1. Variances from MA plots of microarray data[1] (cont'd.)

Polysomal vs. total mRNA comparisons	
HP_{4G} vs. T_{4G}	
19. I vs. I	0.16
20. II vs. II	0.30
21. III vs. III	0.14
	$\overline{X}$ = 0.20
HP_{WT} vs T_{WT}	
22. I vs. I	0.30
23. II vs. II	0.36
24. III vs. III	0.61
	$\overline{X}$ = 0.43

[1]The variances (σ^2) of *M* were calculated as in Fig. 3; $\overline{X}$, mean variances calculated from the values listed in the rows immediately above in that section of the table.

Legend: The comparisons of biological replicates from the same strain, WT or eIF4G mutant, yielded relatively low σ^2 values for both HP and total RNA samples, ranging (with one exception) from 0.03 to 0.17 (rows 1-12). The variances of *M* in plots that compare the intensities of HP mRNAs between mutant and WT cells were 2- to 5-fold higher than the corresponding values for HP replicates from the same strain (rows 13-15 versus 1-6). Similar findings were made for total RNA (rows 16-18 versus 7-12). These comparisons reveal significant differences in the intensities of most mRNAs between mutant and WT cells, for both total and polysomal samples. MA plots that compare intensities in HP versus total RNAs were constructed to quantify the variation in translational efficiency (HP/T) across the genome for each strain. The σ^2 values for the HP:T intensity ratios are, on average, ~2-fold higher for WT than for mutant cells (rows 22-24 vs. 19-21), providing evidence that the range of translational efficiencies (HP/T values) is reduced genome-wide by depletion of eIF4G.

Table S2. PCR Primers for measuring polysome distributions of selected mRNAs

Primer name	Primer Description	Sequence (5' to 3')
FZP261	RPL41A Sense	CGAAATGAGAGCCAAGTGG
FZP262	RPL41A Anti-sense	ATGCAATTTAGATCCATTATGAGG
FZP263	RPL41B Sense	CGACTTAATTCCAAATGAGAGC
FZP264	RPL41B Anti-sense	GATGACGGTCCAATCAAAGG
FZP267	HAC1 Sense	TTACTGAACAGCGTCAAC
FZP268	HAC1 Anti-sense	AGAGGTGAAGGTGTGAAC
FZP271	HSP82 Sense	AGCAACGACGACGAACAATAC
FZP272	HSP82 Anti-sense	GCCACGAACTCAGAATGTCTC
FZP228	ACT1 Sense	TGTGTAAAGCCGGTTTTGCC
FZP229	ACT1 Anti-sense	GATACCTCTCTTGGATTGAGCTTC
FZP275	PDC1-2 Sense	TCTTGGCTGATGCTTGTTGTTC
FZP276	PDC1-2 Anti-sense	GCTTCCTTAACTTCTGGCTTGG
FZP281	18S rRNA Sense	TCACCAGGTCCAGACACAATAAG
FZP282	18S rRNA Anti-sense	TCTCGTTCGTTATCGCAATTAAGC

Table S3. Primers for qRT-PCR determination of TE_{4G}/TE_{WT} ratios and comparison of mean TE_{4G}/TE_{WT} ratios determined by microarray versus qRT-PCR analysis

Primer Pair (sense/ antisense)	ORF/ Gene	Sense Primer (5' to 3')	Antisense Primer (5' to 3')	Average TE_{4G}/TE_{WT} from microarray	Average TE_{4G}/TE_{WT} from qRT-PCR
FZP283/284	*YDL037C/ BSC1*	GCCACTCACA AACGGATTTC	AGGATGAAGT AGTAGAGGAT GAAG	2.5 ± 0.5	0.50
FZP285/286	*YMR317W/ Putative Protein*	GGCAATAGTT CTACCGTTAC AAC	ACAATCAGCG AGGCACTTG	2.1 ± 0.32	1.43 ± 0.19
FZP287/288	*YIL126W/ STH1*	TGATGAATAA TACACCTACA ACAG	TGCCTTGATC TCCAACTTG	2.2 ± 0.80	1.55 ± 0.25
FZP289/290	*YLR403W/ SFP1*	CCAACTACAG GTTCTCACAA CTAC	CATCGTCATC GTCATCGTCA TC	1.6 ± 0.29	1.51 ± 0.17
FZP291/292	*YMR224C/ MRE11*	GCCTTGCGAG TTAGAATTAT TG	TGAAGTATAT CCATAGGACA CAAC	1.5 ± 0.07	1.29 ± 0.26
FZP293/294	*YMR227C/ TAF7/TAF6 7*	ACAAGGAGC AACAACAAG AGGAAG	CCGTCGTCAT CATCGTCATC ATC	1.9 ± 0.7	1.33 ± 0.16
FZP295/296	*YAL019W/ FUN30*	GGAAGACGA TGACGACGAC AATG	GGTGCTGGTG CTGGAGTAGG	1.9 ± 0.73	1.39 ± 0.19
FZP297/298	*YER085C/ Putative Protein*	GAGAGACTGT TTAGCGAGAA TCC	ACCTTCCACA TATCGTCCAA TTC	1.5 ± 0.08	1.41 ± 0.17
FZP299/300	*YOR304W/ ISW2*	CAAACTCCGC AGCCAAAAG C	TCTCCTTCTTC TTCTTCAGCA TCG	1.8 ± 0.57	1.45 ± 0.22
FZP301/302	*YDR138W/ HPR1*	GAAGAGGGC ACTGGAAGA AGAG	CGGCAACACC ACTGTCAGG	1.8 ± 0.58	1.45 ± 0.17
FZP303/304	*YNL308C/ KRI1*	ACCTCTGCGA CGACAGTTAA G	ATCTTCCTCC TCTTCTTCCTC TTC	1.6 ± 0.47	1.09 ± 0.15
FZP307/308	*YDR052C/ DBF4*	ACAACCACAG AATCTCCAAC AG	GCGAAAGAC AAATGCTTCT CAG	1.8 ± 0.71	1.21 ± 0.13
FZP309/310	*YDR228C/ PCF11*	AACTCTCAAT GCTCTGAAAC AAG	TGTGTAAGAA CCGACCATAT CC	1.6 ± 0.37	1.07 ± 0.06
FZP311/312	*YOR219C/ STE13*	CAAGAAGGT AACGGAAGA TGG	CATATTTGTG TAACGGTTTA ATCG	1.5 ± 0.30	1.05 ± 0.13

Primer Pair (sense/ antisense)	ORF/ Gene	Sense Primer (5' to 3')	Antisense Primer (5' to 3')	Average TE_{4G}/TE_{WT} from microarray	Average TE_{4G}/TE_{WT} from qRT-PCR
FZP313/314	*YKL086W/ SRX1*	ACCAACAGA AATTCCTCTC TCAG	GCCACCATTG CGTCGATC	0.58 ± 0.07	0.56 ± 0.08
FZP315/316	*YMR271C/ URA10*	CCTTGAACTG GGATTAGAAT GC	CGAATTTAAG CTCCGATTGA ATG	0.58 ± 0.05	0.55 ± 0.06
FZP317/318	*YER018C/ SPC25*	TGATTCACGC TGCTGGATC	GTGTTGCCCG ACTCTTGG	0.60 ± 0.12	0.59 ± 0.06
FZP319/320	*YHL006C/ SHU1*	GGAGACACTG CCAGGAAG	ATTAGAGTAT TGCGGACCAT TC	0.61 ± 0.18	0.65 ± 0.07
FZP321/322	*YNR037C/ RSM19*	GACTAAGGGC ACTCCAATAA G	TTTGTTCTTG GTTTGAGTAT AGC	0.61 ± 0.14	0.60 ± 0.05
FZP323/324	*YNL327W/ EGT2*	CACTCTCATC CTCATCTGTT C	ACTTGTTCCT GTTATGTTAA TCG	0.64 ± 0.13	0.82 ± 0.11
FZP325/326	*YJR011C/ Hypothetical Protein*	GCAGGAAGA CGAAGTATTA AACC	GACTAGAACC TTCTCGATCA AATG	0.66 ± 0.15	0.62 ± 0.08
FZP327/328	*YGR248W/ SOL4*	CTCGGATGCG GAGAAGATG	CCTCGGCTAC TACTGCTAAT G	0.66 ± 0.17	0.47 ± 0.06
FZP331/332	*YIL157C/ COA1*	ACGGTGAACT GAACTCTGTG AAG	CGGCAGTGAC GCTCCATTC	0.64 ± 0.09	0.56 ± 0.06
FZP333/334	*YDR493W/ AIM8*	TGCGTTCAGG AATGTTATGT C	TCGGTGTCTT TGTGAATGTT C	0.65 ± 0.04	0.48 ± 0.06
FZP335/336	*YJL011C/ RPC17*	CAGGAAGAT GAAGGCGAG GAAC	GGTGGACCAT ATTGGCAGGA AG	0.66 ± 0.07	0.53 ± 0.04
FZP337/338	*YBR230C/ OM14*	CACCACCACA ACAATAAGA AGG	TGACCAAGAC AACGGGATTC	0.65 ± 0.11	0.45 ± 0.06
FZP339/340	*YGR205W/ ATP-Binding Protein*	GGATAGTATT CACCACCGAT AAC	CGTCAATGTA GCAATGGAAC C	0.66 ± 0.10	0.45 ± 0.05
FZP341/342	*YOL026C/ MIM1*	TGACGAATCA GAAGACAAA GAC	GATGGAGCAC GAGCCTAC	0.66 ± 0.07	0.55 ± 0.09
FZP343/344	*YBL018C/ POP8*	ACTCGTCGGT TCACCATTAA CAG	CATAGGCGGT CATCGTCAGT AAC	0.61± 0.11	0.50 ± 0.06

REFERENCES

1. Nagalakshmi U, Wang Z, Waern K, Shou C, Raha D, Gerstein M, Snyder M: **The transcriptional landscape of the yeast genome defined by RNA sequencing**. *Science* 2008, **320**(5881):1344-1349.
